# Ectoparasite Infestations in Wild Mammals from the Lake Van Basin, Eastern Türkiye, with Notes on New Host Records

**DOI:** 10.3390/insects17080807

**Published:** 2026-08-04

**Authors:** Ali Bilgin Yılmaz, Milad Afşar, Muhammed Yasul, Mahsa Torkamanian Afshar, Adnan Ayan, Özge Oktay Ayan, Yaşar Göz, Loğman Aslan

**Affiliations:** 1 Faculty of Health Sciences, Van Yüzüncü Yıl University, Tuşba, Van 65080, Türkiye; yasargoz@yyu.edu.tr; 2 Division of Medical Parasitology, Department of Basic Medical Sciences, Faculty of Medicine, Van Yüzüncü Yıl University, Tuşba, Van 65080, Türkiye; mtamilad@gmail.com (M.A.); ozgeokty09@gmail.com (Ö.O.A.); 3 Home Health Care Program, Health Care Services Department, Bartın University School of Health Services, Bartın 74100, Türkiye; myasul@bartin.edu.tr; 4 Department of Software Engineering, Faculty of Engineering and Natural Sciences, İstanbul Topkapı University, İstanbul 34020, Türkiye; mahsatorkamanianafshar@topkapi.edu.tr; 5 Faculty of Veterinary Medicine, Aksaray University, Aksaray 68100, Türkiye; adnan.ayan@aksaray.edu.tr; 6 Wildlife Protection and Rehabilitation Center, Van Yüzüncü Yıl University, Tuşba, Van 65080, Türkiye; logmanaslan@yyu.edu.tr

**Keywords:** wildlife parasites, fleas, ticks, zoonotic diseases, biodiversity, Lake Van Basin

## Abstract

Ectoparasites cause skin lesions, anemia, and mortality in wild animals and act as vectors of zoonotic pathogens. This study reports ectoparasites detected during an opportunistic survey on wild mammals admitted to the Van Yuzuncu Yil University Wildlife Protection and Rehabilitation Center in eastern Türkiye during 2024–2025. Due to the opportunistic nature of sampling, the findings primarily reflect parasite burdens in injured or debilitated individuals and may not be representative of healthy free-ranging populations. Of the 28 animals examined, 20 (71.4%) were infested. A total of 449 ectoparasites were collected: 370 fleas (82.4%), 58 ticks (12.9%), and 21 lice (4.7%). Identified species included four fleas (*Paraceras melis*, *Chaetopsylla globiceps*, *Pulex irritans*, *Archaeopsylla erinacei*), four ticks (*Ixodes kaiseri*, *Rhipicephalus turanicus*, *Haemaphysalis parva*, *Haemaphysalis erinacei*), and one louse (*Trichodectes melis*). This study provides new host–ectoparasite association records for Türkiye: *I. kaiseri* and *R. turanicus* on beech marten (*Martes foina*); *I. kaiseri* and *C. globiceps* on Eurasian badger (*Meles meles*); and *P. irritans* on Eurasian lynx (*Lynx lynx*) and brown bear (*Ursus arctos*). Bipartite network analysis revealed moderate specialization (H2’ = 0.52) and marked variation in host specificity, with *T. melis*, *A. erinacei*, and *P. melis* showing high host specificity (d’ > 0.85), whereas *Chaetopsylla globiceps* and *Pulex irritans* acted as generalists (d’ < 0.30). Some identified ectoparasites are known or suspected vectors of zoonotic pathogens. These findings provide baseline data for future research on wildlife health and disease transmission risks in the region.

## Simple Summary

Wild mammals can carry small creatures called ectoparasites—such as fleas, ticks, and lice—that live on their bodies. Some of these ectoparasites can cause diseases in animals and sometimes even in humans. In this study, we examined 28 wild mammals (badgers, martens, bears, lynxes, foxes, and hedgehogs) that were brought to a wildlife rehabilitation center in eastern Türkiye. We found that 20 of the 28 animals (71.4%) were infested with ectoparasites. In total, we collected 449 ectoparasites: 370 fleas, 58 ticks, and 21 lice. We identified nine different species, with five of these representing new host–parasite associations (new host records) for Türkiye. Using a special network analysis, we discovered that some parasites were very picky about which host they live on, while others (especially two flea species) were generalists that can live on many different hosts. These generalist fleas are important because they could potentially spread pathogens between wild animals, domestic animals, and humans. This study provides the first basic information about ectoparasites in this region and will help scientists understand the risk of disease transmission in the future.

## 1. Introduction

Heavy ectoparasite infestations can lead to direct clinical conditions, including anemia, allergic dermatitis, skin necrosis, secondary bacterial infections, reduced reproductive fitness, and increased mortality [1,2]. Beyond their direct parasitic effects, ectoparasites are of major medical and veterinary concern due to their role as reservoirs and vectors for a wide range of zoonotic pathogens [3].

Among ectoparasites, ticks (suborder Ixodida), fleas (order Siphonaptera), and lice (order Phthiraptera) are of particular importance [4]. Ticks are key vectors of zoonotic bacteria, viruses, and protozoa [5], while fleas transmit pathogens such as *Yersinia*, *Rickettsia*, and *Bartonella* spp., and lice are known vectors for agents causing swinepox and rickettsial infections [6].

Türkiye’s unique geographical position at the junction of Europe and Asia, combined with its diverse habitats, supports a rich mammalian fauna, with 177 species recorded nationally [7]. The Lake Van Basin, in eastern Türkiye, is a region of notable biodiversity, hosting endemic species such as the pearl mullet (*Alburnus tarichi*) and meeting eight of the nine Ramsar Convention criteria for wetlands of international importance [8]. Despite this diversity and the recognized importance of ectoparasites in disease ecology, studies on ectoparasites of wild mammals in Türkiye, particularly in the Lake Van Basin, remain scarce. To our knowledge, only one study has examined ectoparasites in hedgehogs from Van Province [9], and no systematic survey exists for other wild mammal species in the basin.

We explored the hypothesis that fossorial or den-dwelling species (e.g., badgers) support higher ectoparasite richness and diversity compared to wide-ranging carnivores (e.g., lynx, bear), and predicted that network analysis would reveal a gradient of host specificity from host-specific lice and hedgehog fleas to generalist fleas. However, due to the small and opportunistic sample, these hypotheses are explored descriptively rather than tested statistically. To test this hypothesis, the present study aimed to report new host–parasite associations across several wild mammal species and document ectoparasite diversity in wild mammals admitted to a wildlife rehabilitation center in the Lake Van Basin, eastern Türkiye. This study represents the first systematic ectoparasite survey of wild mammals in the Lake Van Basin. By combining traditional morphological identification with bipartite network analysis, we provide both a taxonomic inventory and an ecological framework for understanding host–parasite community structure in this understudied region.

## 2. Materials and Methods

### 2.1. Study Area and Sample Collection

**Figure 1:**
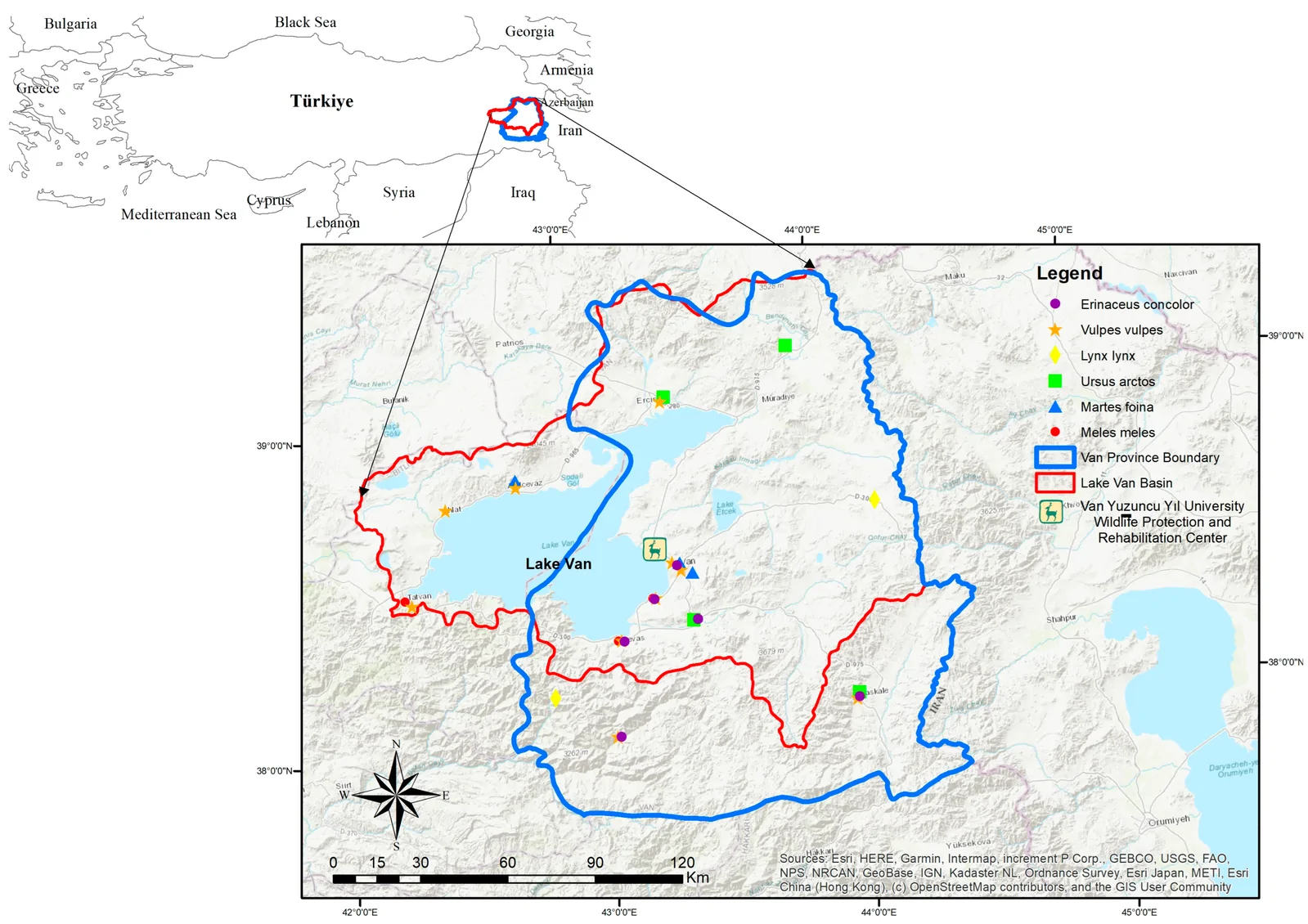
Map of the Lake Van Basin, eastern Türkiye, showing the study area and approximate rescue locations of wild mammals examined in this study. Symbols indicate host species: red circles = *Erinaceus concolor*, orange stars = *Vulpes vulpes*, yellow diamonds = *Lynx lynx*, green squares = *Ursus arctos*, blue triangles = *Martes foina*, purple circles = *Meles meles*. The dashed line indicates the Van Province boundary, and the shaded area indicates the Lake Van Basin. The red star marks the location of Van Yüzüncü Yıl University Wildlife Protection and Rehabilitation Center. The inset map shows the location of the study area within Türkiye. Scale bar = 50 km.

The study was conducted at the Van Yuzuncu Yıl University Wildlife Protection and Rehabilitation Center in Van province, eastern Türkiye, between January 2024 and December 2025 (Figure 1). All animals were rescued from various localities within the Lake Van Basin, including districts of both Van and Bitlis provinces. A total of 28 wild mammals from six species (*Meles* meles (n = 3), Martes foina (n = 2), Ursus arctos (n = 4), Lynx lynx (n = 2), Vulpes vulpes (n = 7), and Erinaceus concolor (n = 10) admitted due to injury, vehicular collision, or debilitation were examined for ectoparasites. All animals were admitted due to injury, vehicular collision, or debilitation; thus, the sampled population may not fully represent healthy, free-ranging individuals. Ectoparasites were collected through comprehensive manual examination of the entire body surface, with special attention to the head, ears, axillae, and inguinal regions. For large carnivores (bears and lynxes), collection was performed under sedation (ketamine 5 mg/kg + xylazine 1 mg/kg), whereas physical restraint sufficed for other species. Sedation was preferred to minimize animal stress and ensure welfare, to guarantee the safety of both the animals and the research team, and to enable thorough and safe examination of the entire body surface, including hard-to-reach areas. This approach is in accordance with the animal ethics committee approval (E-27552122-604.01-767300).

### 2.2. Parasite Processing and Identification

Collected ectoparasites were preserved in 70% ethanol. Fleas and lice were cleared in 10% KOH for 24 h, dehydrated through a graded ethanol series (70–100%), cleared in xylene, and mounted on slides with Canada balsam. Ticks were examined under a stereomicroscope without clearing. Species identification followed standard taxonomic keys for fleas [10,11,12], ticks [13,14,15] and lice [16]. Although some taxonomic keys are dated, they remain the standard references for morphological identification of these taxa in the Palearctic region. Representative specimens were photographed using a stereomicroscope (SOIF ST8050-B8LS, Shenzhen Zhongzheng Instruments Co., Ltd., Shenzhen, China) and a light microscope (Leica DM750, Leica Microsystems, Wetzlar, Germany), each equipped with a digital imaging analysis system (MSHOT, Guangzhou Micro-shot Technology Co., Ltd., Guangzhou, China; V1.1.6). Micrographs were taken at magnifications of 40× to 400× depending on parasite size.

### 2.3. Definitions and Statistical Analysis

Prevalence was defined as the percentage of infested hosts within each species. Mean infestation intensity was calculated as the total number of ectoparasites collected from infested individuals divided by the number of infested hosts. Prevalence (%) was calculated with 95% confidence intervals using the Wilson score method. Mean infestation intensity and standard deviation were calculated for each host species. Bipartite network analysis was performed using the bipartite package (version 2.18) in R (version 4.3.1). Network metrics included connectance (C), H2’ specialization index [17], modularity (QuanBI), host specificity index (d’) at the parasite level, and interaction diversity (Shannon H’) at the host level. Null model comparisons (n = 1000 randomizations) were used to assess whether observed metrics differed significantly from random expectations (*p* > 0.05 for all metrics), indicating that the observed patterns should be interpreted cautiously given the limited sample size.

### 2.4. Data and Material Deposition

All voucher specimens are deposited in the personal collection of the corresponding author at Van Yuzuncu Yil University, Faculty of Health Sciences, Van, Türkiye. Upon acceptance of the manuscript, specimens will be transferred to an institutional collection (Van Yüzüncü Yıl University Zoology Museum) with final accession numbers (temporary accession numbers: VOUCH-2024-001 to VOUCH-2024-071).

**Figure 2:**
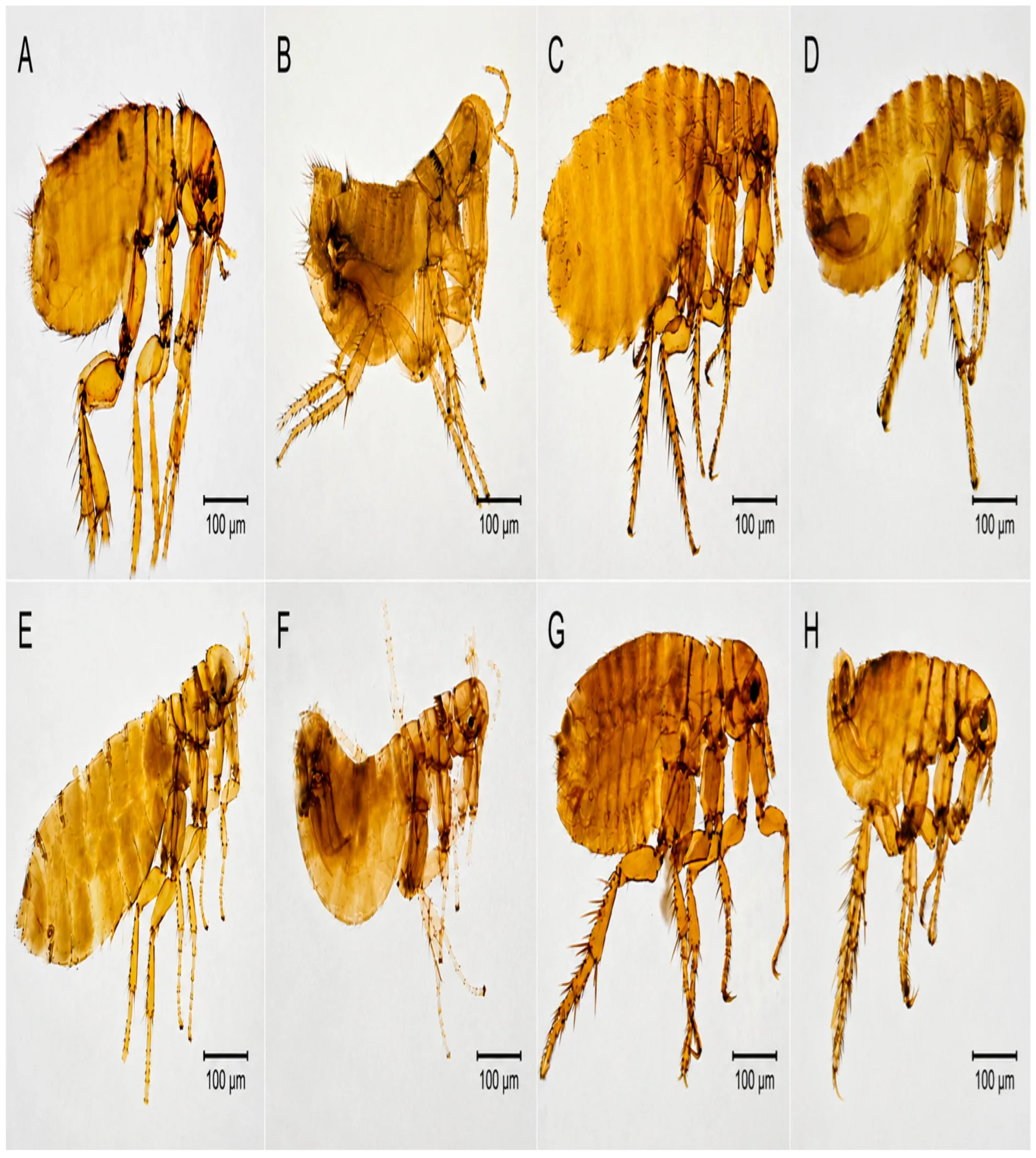
Flea species identified in this study. Scale bar = 100 µm. (**A**) *A. erinacei* (female), (**B**) *A. erinacei* (male), (**C**) *C. globiceps* (female), (**D**) *C. globiceps* (male), (**E**) *P. melis* (female), (**F**) *P. melis* (male), (**G**) *P. irritans* (female), (**H**) *P. irritans* (male).

**Figure 3:**
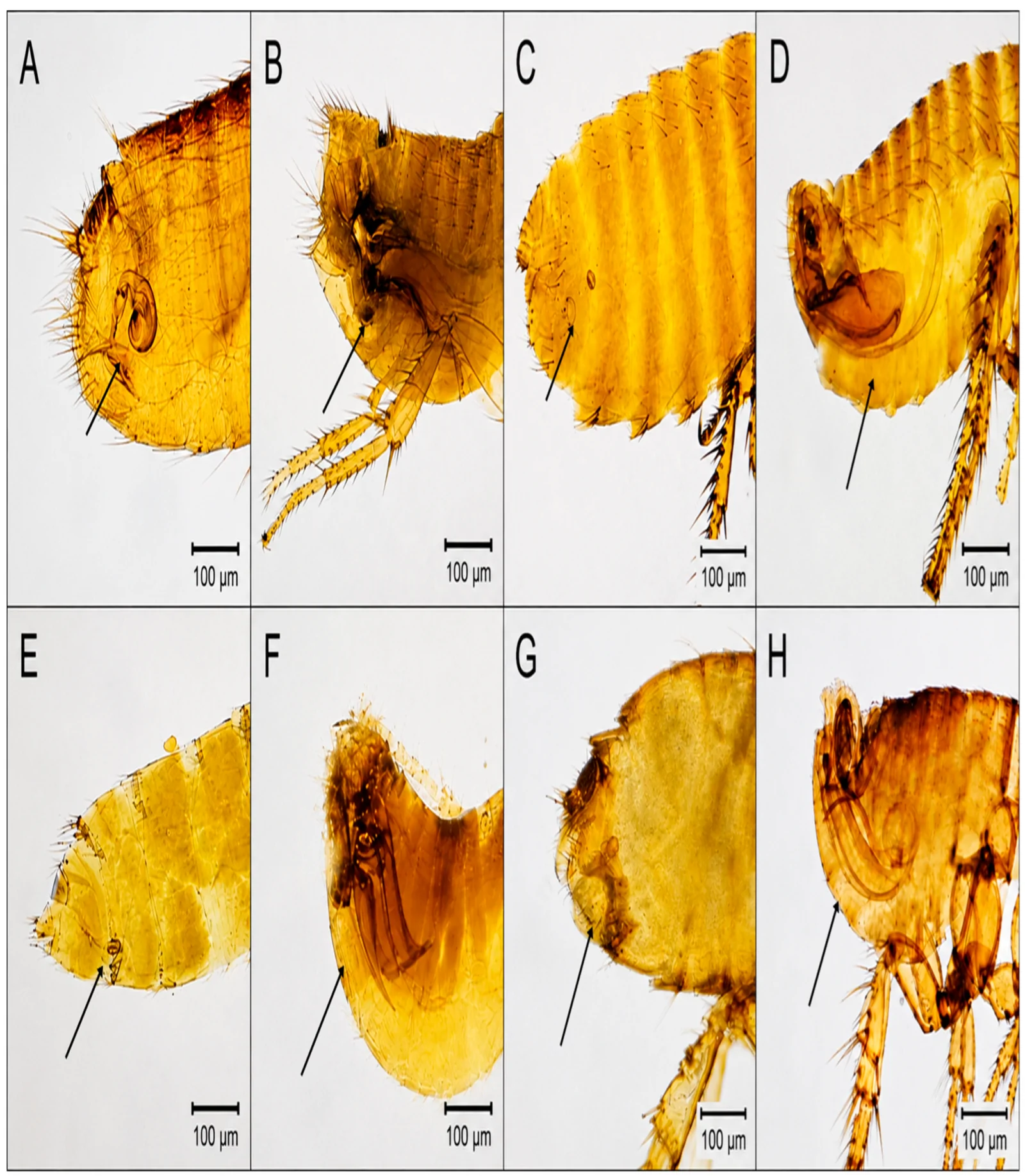
The arrows in figures (**A**,**C**,**E**,**G**) point to the spermathecal capsules of the female fleas. The arrows in figures (**B**,**D**,**F**,**H**) point to the claspers of the male fleas.

**Figure 4:**
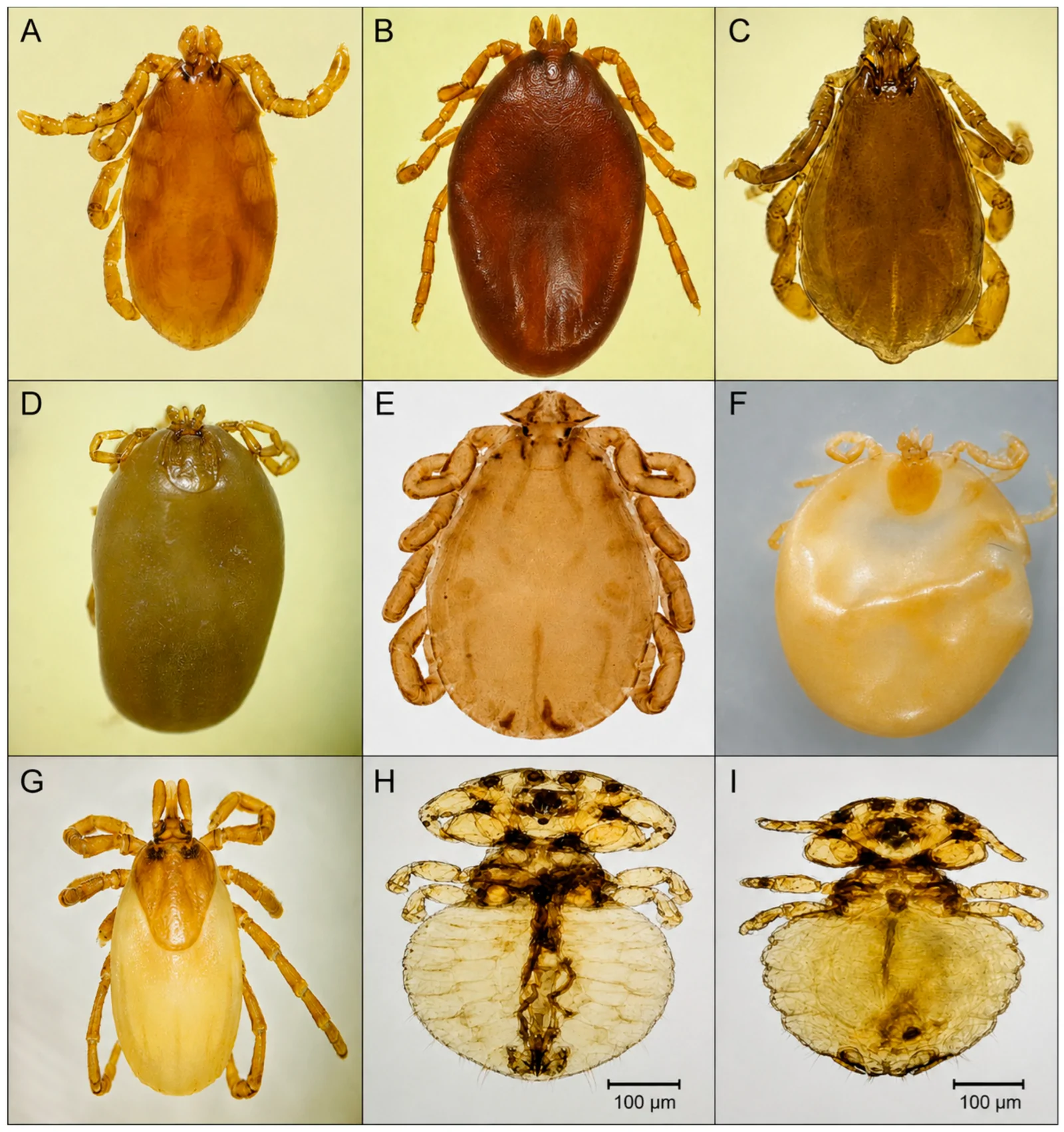
Lice and ticks identified in this study. Scale bars = 100 µm for (**A**) *Hae. parva* (male), (**B**) *Hae. parva* (female), (**C**) *R. turanicus* (male), (**D**) *R. turanicus* (female), (**E**) *Hae. erinacei* (male), (**F**) *Hae. erinacei* (female), (**G**) *I. kaiseri* (female), 100 µm for (**H**,**I**) *T. melis* (male–female).

**Figure 5:**
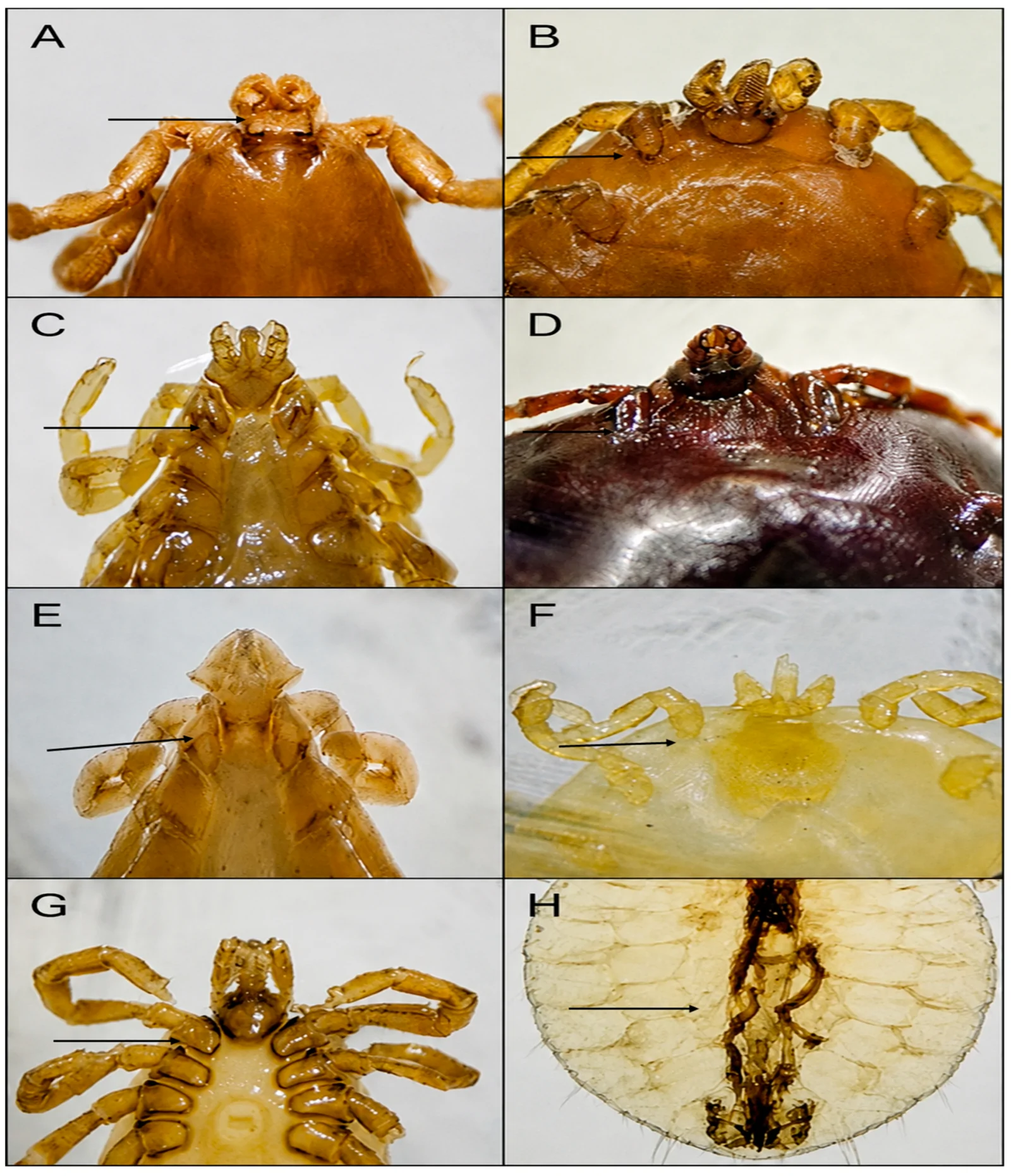
The arrows in figure (**A**) point to the capitulum of the male tick. The arrows in figures (**B**–**G**) point to Coxa I of female and male ticks, and (**H**) *T. melis* male terminalia.

Diagnostic characters used for identification are documented in the photographic plates (Figure 2, Figure 3, Figure 4 and Figure 5).

## 3. Results

Ectoparasite infestation was detected in 20 out of 28 examined wild mammals, corresponding to a prevalence of 71.4% (95% CI: 51.2–86.4%). A total of 449 ectoparasite specimens were collected, comprising 370 fleas (82.4%), 58 ticks (12.9%), and 21 lice (4.7%). Nine distinct species were identified: four fleas, four ticks, and one louse. Detailed prevalence and infestation intensity data for each host species are presented in Table 1.

The high standard deviations relative to mean intensities, particularly in badgers (SD = 31.5, mean = 47.0), may suggest substantial individual variation in parasite loads within host species. However, given the small sample sizes (n = 3 for badgers), this variation could reflect the influence of a single heavily infested individual rather than true parasite aggregation. These observations are preliminary and require confirmation with larger sample sizes.

Prevalence rates varied markedly among host species, ranging from 50% in lynx to 100% in badgers, martens, and foxes. Total ectoparasite loads also differed substantially across species, with badgers exhibiting the highest burden and martens the lowest (Table 1).

From badgers, *Trichodectes melis* (21 specimens) (Figure 4H,I), *Paraceras melis* (35), *Chaetopsylla globiceps* (75), and *Ixodes kaiseri* (10) (Figure 5G) were collected. From martens, one female *I. kaiseri* and 18 *Rhipicephalus turanicus* (11 females, 7 males) (Figure 5C,D) were collected. From bears, 30 *Pulex irritans* and three *Haemaphysalis parva* (Figure 5A,B) were collected. The single infested lynx carried 33 *P. irritans* specimens. From foxes, *C. globiceps* (78 specimens; 32 males, 46 females), *P. irritans* (52; 29 males, 23 females), and *Haemaphysalis erinacei* (12; 6 males, 6 females) (Figure 5E,F) were collected. From hedgehogs, *Archaeopsylla erinacei* (67; 29 males, 38 females) and *R. turanicus* (14; 3 males, 11 females) were collected.

Representative micrographs of the identified flea species are shown in Figure 2 (*P. melis*, *C. globiceps*, *P. irritans*, *A. erinacei*). Figure 3 highlights the spermathecal capsules of female fleas (A, C, E, G) and the claspers of male fleas (B, D, F, H). Figure 4 presents the louse and tick species identified in this study, including *T. melis*, *I. kaiseri*, *R. turanicus*, *Hae. parva*, and *Hae. erinacei*. Figure 5 shows key diagnostic structures of ticks, including the capitulum (A), Coxa I (B, C, D, E, G), and male terminalia (H).

To characterize the structure of host–parasite interactions beyond species-level prevalence, we performed a bipartite network analysis. The overall connectance was 0.306, indicating that only 30.6% of all possible host–parasite links were observed (Table 2). The H2’ specialization index (0.52) suggested an intermediate level of host specificity in this system. Among parasites, *T. melis*, *A. erinacei*, and *P. melis* exhibited the highest host specificity (d’ > 0.85), each restricted to a single host species. In contrast, *C. globiceps* and *P. irritans* showed low specificity (d’ = 0.28 and 0.24, respectively), infesting multiple distantly related hosts. Among hosts, badgers (*M. meles*) had the highest interaction diversity (H’ = 0.94), while lynx (*L. lynx*) had the lowest (H’ = 0.00) (Table 2).

## 4. Discussion

In this study, we documented a high overall prevalence of ectoparasites (71.4%; Table 1) among wild mammals admitted to a rehabilitation center in eastern Türkiye, with fleas accounting for 82.4% of all collected specimens. The predominance of fleas is likely related to the shelter-use habits of the host species in the Lake Van Basin. Although true fossorial behavior is evident only in badgers (*M. meles*), the enclosed shelters used by other hosts (e.g., dens, cavities, abandoned burrows) provide suitable microclimatic conditions for the nidicolous (nest-dwelling) life cycle of fleas, facilitating population establishment independent of direct burrowing behavior [18,19].

The presence of *C. globiceps* on badgers is noteworthy. While *C. globiceps* is a common parasite of red foxes across its range [11,20,21,22], records from badgers are extremely scarce, with only one historical report outside Türkiye [23]. To our knowledge, this study provides the first documented infestation of *M. meles* by *C. globiceps* in Türkiye. A possible explanation is ecological overlap in den use between badgers and foxes, which could facilitate cross-species flea transfer. However, this hypothesis is not directly tested here, and direct concurrent den use is not well documented in the literature. Ectoparasites can persist in vacant burrows for variable periods, potentially allowing transmission without direct host contact. Similarly, *I. kaiseri*, typically reported from hedgehogs and foxes [13], was found on *M. meles* in this study, representing a new host record for Türkiye and consistent with occasional reports from other regions [14,16]. Given the small sample size (n = 3), these interpretations remain preliminary.

Similar to badgers, mustelids also harbored previously unreported ectoparasite associations. The detection of *I. kaiseri* on *M. foina* constitutes the first record of this tick species on beech marten in Türkiye, although *I. kaiseri* has been previously reported from foxes in the country [24]. Additionally, while *R. turanicus* is a widely distributed tick infesting domestic ruminants and dogs, with immature stages often found on small mammals and hedgehogs [15], and has been reported from *M. foina* in Spain [25], it has not been recorded from this host in Türkiye [26]. Thus, the presence of *R. turanicus* on beech marten is reported as another new host record for Türkiye. These findings suggest that mustelids may contribute to the ecology of these tick species, but larger sample sizes are needed to confirm this pattern.

Beyond mustelids, large carnivores in this study also revealed new host records. The human flea, *P. irritans*, was found on both brown bear (three of four individuals) and Eurasian lynx (one of two individuals). To the best of our knowledge, this study presents the first documented cases of *P. irritans* infestation on *U. arctos* and *L. lynx* in Türkiye. Previous studies have reported this flea on the American black bear (*Ursus americanus*) in the USA [27] and on the Eurasian lynx in Europe [25,28], supporting the plausibility of such associations. It is hypothesized that large, wide-ranging carnivores may play a role in the dispersal of fleas across landscapes, although this remains to be tested. The finding of *Hae. parva* on bears is consistent with other reports from Türkiye, where this tick parasitizes various wild carnivores [29]. Due to the very small sample sizes (bears n = 4, lynx n = 2), these findings should be considered anecdotal and require confirmation in larger populations.

In contrast to these novel associations, our findings from red foxes and hedgehogs corroborated previously documented host–parasite relationships in Türkiye. From foxes, we collected (see Table 1 for detailed counts) *C. globiceps*, *P. irritans*, and *Hae. erinacei*, all of which have been previously reported from this host in the country [11,24,30,31]. From hedgehogs, the hedgehog flea *A. erinacei* and the tick *R. turanicus* were collected, confirming their status as common parasites of *E. concolor* in Türkiye [9,31,32]. The relatively high infestation rate of *A. erinacei* (60% prevalence; Table 1) suggests its local abundance, though confirmation with larger sample sizes is warranted. Beyond confirming these known associations, the bipartite network analysis (see below) allowed us to quantify host specificity and interaction diversity across the entire parasite community.

### Host–Parasite Network Structure

The bipartite network analysis provided insights into the overall structure of ectoparasite communities in this system (Table 2). The connectance value of 0.306 indicates that only 30.6% of all possible host–parasite links were realized. The intermediate H2’ specialization index (0.52) positions this host–parasite network between fully generalized and highly specialized systems. At the parasite level, host specificity indices (d’) revealed a clear dichotomy: *T. melis* (0.92), *A. erinacei* (0.88), and *P. melis* (0.85) showed very high specificity, each restricted to a single host species, whereas *C. globiceps* (0.28) and *P. irritans* (0.24) exhibited low specificity, infesting multiple distantly related hosts. At the host level, badgers displayed the highest interaction diversity (H’ = 0.94), while lynx showed the lowest (H’ = 0.00) (Table 2).

Notably, the two generalist flea species in our network (*C. globiceps* and *P. irritans*) exhibited low host specificity (d’ < 0.30; Table 2), which may increase their potential to bridge pathogen transmission across multiple host species, including domestic animals and humans. Several ectoparasite species identified in this study are of considerable zoonotic importance. *P. irritans*, *I. kaiseri*, and *R. turanicus* have been reported as known or suspected vectors of bacterial pathogens (e.g., *Rickettsia* spp., *Borrelia* spp.) and other disease agents [4,33,34]. The detection of these ectoparasites on wild mammals in the Lake Van Basin highlights the potential importance of including wildlife in regional disease surveillance. However, no direct pathogen analysis was conducted in this study, and the actual vectorial role of these ectoparasites in the region remains to be investigated. Heavy ectoparasite loads can also directly affect host fitness, leading to anemia, dermatitis, and secondary infections [2,35].

Several limitations must be acknowledged. Due to the modest sample size, inferential statistical tests (e.g., chi-square, Kruskal–Wallis) were not performed, and our network metrics should be interpreted as descriptive rather than confirmatory. First, the sample size was modest (n = 28), and all specimens were obtained from injured, debilitated, or orphaned animals admitted to a rehabilitation center. The high standard deviations observed in mean intensity (Table 1) indicate substantial individual variation in parasite loads, which is typical for wildlife parasite aggregation but also limits the precision of our prevalence estimates. Such individuals may not fully represent the parasite burdens of healthy, free-ranging populations due to potential differences in behavior, immune status, or microhabitat use. Second, the absence of seasonal sampling prevents assessment of temporal variation in ectoparasite abundance and diversity. Third, this study did not include molecular screening for pathogen DNA/RNA, limiting conclusions regarding zoonotic risk. Finally, the interpretation of new host records, particularly for species with small sample sizes (e.g., lynx, marten), should be considered preliminary.

Future studies should prioritize systematic, seasonal sampling of free-ranging wildlife populations across broader geographic areas in eastern Türkiye. Complementary molecular characterization of ectoparasites and their associated pathogens (e.g., using next-generation sequencing or targeted PCR) is essential to assess actual vector competence and zoonotic potential. Long-term monitoring at the wildlife–human–livestock interface would be particularly valuable given the increasing habitat overlap in the region.

## 5. Conclusions

This opportunistic survey provides the first baseline data on ectoparasite diversity in wild mammals admitted to a rehabilitation center in the Lake Van Basin, identifying nine species across three arthropod taxa and documenting several new host records for Türkiye. These records expand our knowledge of host–parasite associations in the region; however, due to the small and non-random sample, these findings should be considered preliminary and require confirmation through systematic surveys of free-ranging populations. Bipartite network analysis suggested moderate specialization (H2’ = 0.52) and indicated a preliminary distinction between parasites observed on a single host (*T. melis*, *A. erinacei*, *P. melis*) and those found on multiple hosts (*C. globiceps*, *P. irritans*) in this limited dataset. These descriptive patterns require validation with larger samples from free-ranging populations. Importantly, these patterns do not necessarily reflect true host specificity and should be validated with larger samples. The presence of potential vectors of zoonotic pathogens, particularly the generalist flea species, highlights the need for integrated surveillance at the wildlife–domestic animal–human interface. Future research should prioritize systematic, seasonal sampling of free-ranging wildlife populations, incorporate molecular diagnostics to assess pathogen presence and vector competence, and include voucher deposition in accessible institutional collections to support taxonomic verification.

## Figures and Tables

**Table 1:** Prevalence and mean infestation intensity of ectoparasites in wild mammals from eastern Türkiye. CI: confidence interval (Wilson score method); SD: standard deviation. For *Lynx lynx*, only one infested individual was detected; therefore, SD could not be calculated. Mean intensity is presented as a raw value for this species.

Host Species	Examined (n)	Infested (n)	Prevalence (%)	95% CI	Total	Mean Intensity ± SD
*Meles meles*	3	3	100	43.9–100	141	47.0 ± 31.5
*Martes foina*	2	2	100	34.2–100	19	9.5 ± 11.3
*Ursus arctos*	4	3	75.0	30.1–95.4	33	11.0 ± 14.9
*Lynx lynx*	2	1	50.0	9.5–90.5	33	33.0 (n = 1)
*Vulpes vulpes*	7	7	100	64.6–100	142	20.3 ± 15.1
*Erinaceus concolor*	10	6	60.0	31.3–83.2	81	13.5 ± 14.7
Total	28	20	71.4	51.2–86.4	449	22.5 ± 19.9

**Table 2:** Bipartite network metrics of host–ectoparasite associations in wild mammals from eastern Türkiye.

Level	Metric	Value	Interpretation
Host–parasite network	Connectance (C)	0.306	30.6% of all possible links realized
	H2’ (specialization)	0.52	Intermediate specialization
	Modularity (QuanBI)	0.38	Weak modular structure
Parasite-level (d’)	*T. melis*	0.92	Observed only on *M. meles* in this dataset
	*A. erinacei*	0.88	Observed only on *E. concolor* in this dataset
	*P. melis*	0.85	Observed only on *M. meles* in this dataset
	*Hae. erinacei*	0.75	Observed on a limited number of hosts
	*I. kaiseri*	0.62	Observed on a limited number of hosts
	*R. turanicus*	0.31	Observed on multiple host species in this dataset
	*C. globiceps*	0.28	Observed on multiple host species in this dataset
	*P. irritans*	0.24	Observed on multiple host species in this dataset
Host-level (H’)	*M. meles*	0.94	Highest parasite diversity
	*V. vulpes*	0.82	High parasite diversity
	*M. foina*	0.60	Moderate parasite diversity
	*U. arctos*	0.59	Moderate parasite diversity
	*E. concolor*	0.43	Low parasite diversity
	*L. lynx*	0.00	Lowest parasite diversity

Note: C: connectance; H2’: Blüthgen specialization index (0 = fully generalized, 1 = fully specialized); d’: host specificity index (d’ > 0.7 = highly specific); H’: Shannon diversity of interactions (higher values indicate greater evenness across parasite species). Calculations were performed using the bipartite package (R) with null model comparisons (n = 1000 randomizations).

## Data Availability

The original contributions presented in this study are included in the article. Further inquiries can be directed to the corresponding author(s).
